# Supplementary material for: Predicting software reuse using machine learning techniques—A case study on open-source Java software systems
Source: PLoS One. 2025 Feb 13;20(2):e0314512. doi: 10.1371/journal.pone.0314512 (PMC11824963; doi:10.1371/journal.pone.0314512)
Supplement: S1 File — (PDF) [file pone.0314512.s001.pdf]

Appendix 1 Classification results: `https://github.com/cyan-wings/software-reuse-thesis/tree/master/Classification`.

1. Firstly, tests were conducted on various scaling or normalisation methods. These tests also considered whether the absence and presence of performing variance threshold or resampling improved the results. Optimal results for all the 11 ML classifiers can be viewed here. Results can be viewed in the following links:

   No variance thresholding and no resampling

   No variance thresholding but with resampling

   Variance thresholding but no resampling

   With variance thresholding and resampling

2. Next, PCA, KBf or RFi feature selection was individually applied to each classifier from the optimal pipeline setting from Step 1 to test whether it could further improve the models' performance. The results of all the models can be viewed here.

3. Each classifier with the optimal feature selection and pipeline setting can be viewed here.

4. Subsequently, permutation importance was performed on each optimal classifier from Step 3. Results for each model can be viewed here. In the PI spreadsheet of every model, the values in the second, third and fourth columns are relevant to the feature's importance score during the permutation importance evaluation for every cross-validation fold instance. The first ten rows represent the top 10 important features of the first cross-validation fold instance, while the next ten rows represent the top 10 important features of the next cross-validation fold instance... There are 40 cross-validation fold instances, leading to 400 rows (important features) in every PI spreadsheet.

5. Each unique important feature from each classifier in Step 4 was then summed based on the F-score of that classifier's cross-validation fold instance. Results of all the unique features with its aggregated score can be viewed here.

6. Finally, unique features of all the models were amassed based on the score observed in Step 5 and labelled based on their category and granularity. The cumulative of all classifiers' important features can be viewed here.

7. Category and granularity scores were also summed based on Step 6. They can be viewed here and here.

Appendix 2 Regression results: `https://github.com/cyan-wings/software-reuse-thesis/tree/master/Regression`.

1. Firstly, tests were conducted on various scaling or normalisation methods. These tests also considered whether the absence and presence of performing variance threshold improved the results. Optimal results for all the 11 ML regressors can be viewed here. Results can be viewed in the following links:

   Variance thresholding

No variance thresholding

2. Next, PCA, KBf or RFi feature selection was individually applied to each regressor from the optimal pipeline setting from Step 1 to test whether it could further improve the models' performance. The results of all the models can be viewed here.
3. Each regressor with the optimal feature selection and pipeline setting can be viewed here.
4. Subsequently, permutation importance was performed on each optimal regressor from Step 3. Results for each model can be viewed here. In the PI spreadsheet of every model, the values in the second, third and fourth columns are relevant to the feature's importance score during the permutation importance evaluation for every cross-validation fold instance. The first ten rows represent the top 10 important features of the first cross-validation fold instance, while the next ten rows represent the top 10 important features of the next cross-validation fold instance... There are 40 cross-validation fold instances, leading to 400 rows (important features) in every PI spreadsheet.
5. Each unique important feature from each regressor in Step 4 was then summed based on the F-score of that regressor's cross-validation fold instance. Results of all the unique features with its aggregated score can be viewed here.
6. Finally, unique features of all the models were amassed based on the score observed in Step 5 and labelled based on their category and granularity. The cumulative of all regressors' important features can be viewed here.
7. Category and granularity scores were also summed based on Step 6. They can be viewed here and here.

Appendix 3 **Source code** that include most of the data pipeline: `https://github.com/cyan-wings/software-reuse-thesis/blob/master/CC-SM-Classification_v3.ipynb` and `https://github.com/cyan-wings/software-reuse-thesis/blob/master/CC-SM-Regression_v3-2.ipynb.ipynb`. A mirror of the repository with a persistant identifier: `https://doi.org/10.5281/zenodo.13766929`.
