# Supplementary material for: Predicting software reuse using machine learning techniques—A case study on open-source Java software systems
Source: PLoS One. 2025 Feb 13;20(2):e0314512. doi: 10.1371/journal.pone.0314512 (PMC11824963; doi:10.1371/journal.pone.0314512)
Supplement: Attachment — Submitted filename: Response to Reviewers.docx [file pone.0314512.s002.docx]

Dear Editors and Reviewers,

# Response to Reviewers

We would like to express our gratitude for reviewing this research work in detail.

The table below observes the response towards the suggestions and comments onto the written manuscript:

| Comment or Concern | Explanation | Changes Made |
| --- | --- | --- |
| 1. Please ensure that your manuscript meets PLOS ONE's style requirements, including those for file naming. The PLOS ONE style templates can be found at  <https://journals.plos.org/plosone/s/file?id=wjVg/PLOSOne_formatting_sample_main_body.pdf> and  <https://journals.plos.org/plosone/s/file?id=ba62/PLOSOne_formatting_sample_title_authors_affiliations.pdf> | Thank you for the reminder. The PlosOne Latex template was used to generate the formatting of the document. Authors are aware of the prior alterations of the format especially in the title, author, affiliations section. This will be reverted based on the template with added skips just to ensure it looks like the one in the given link. Authors are unsure if there needs to be a separate first page for the title, author, affiliations, thus will stick to what is given in the template. | - Changed all ‘Figure’ to ‘Fig’. - Reverted formatting of the first page based on the Latex template. - Headings and subheadings are all changed to sentence case. |
| 2. Please note that PLOS ONE has specific guidelines on code sharing for submissions in which author-generated code underpins the findings in the manuscript. In these cases, all author-generated code must be made available without restrictions upon publication of the work. Please review our guidelines at <https://journals.plos.org/plosone/s/materials-and-software-sharing#loc-sharing-code> and ensure that your code is shared in a way that follows best practice and facilitates reproducibility and reuse. | Thanks for the guidelines. | [DOI: 10.5281/zenodo.13766929](https://doi.org/10.5281/zenodo.13766929)  This link will be included in the Data Availability statement. |
| 3. Please amend your list of authors on the manuscript to ensure that each author is linked to an affiliation. Authors’ affiliations should reflect the institution where the work was done (if authors moved subsequently, you can also list the new affiliation stating “current affiliation:….” as necessary). | - | The authors remain the same. Though formatting is slightly amended based on the Latex template given by PlosOne. |
| 3. Have the authors made all data underlying the findings in their manuscript fully available?  The PLOS Data policy requires authors to make all data underlying the findings described in their manuscript fully available without restriction, with rare exception (please refer to the Data Availability Statement in the manuscript PDF file). The data should be provided as part of the manuscript or its supporting information, or deposited to a public repository. For example, in addition to summary statistics, the data points behind means, medians and variance measures should be available. If there are restrictions on publicly sharing data—e.g. participant privacy or use of data from a third party—those must be specified.  Reviewer #1: Yes  Reviewer #2: No | Dataset link has been publicly available. It is attached as a footnote. Under Section 3.2.1.  <https://github.com/cyan-wings/software-reuse-thesis/blob/master/Classification/Dataset.csv> | - |
| Reviewer #1: I recommend making the source codes publicly available to enhance transparency and reproducibility. | - | Added in the appendix and referenced in Conclusion. |
| Reviewer #1: The paper’s structure could be improved. I suggest reorganizing it into sections: "Research Questions," "Experiment Design," "Experiment Setup," and "Experiment Results," with subsections for "Quantitative Results" and "Qualitative Results." Currently, the research questions are presented as Section 1 of the paper. I recommend just mentioning them briefly in the introduction section and then moving the detailed discussion of the research questions to the proposed approach section.  Reviewer #1: Overall, the paper addresses a crucial problem in software engineering with a well-written manuscript. However, it requires significant restructuring, additional details, and a broader experimental evaluation as well as exploration of modern techniques to enhance its comprehensiveness and impact. | Thank you for your feedback regarding the structure of the paper. We appreciate your suggestions to enhance clarity and organization. We have amended the introduction accordingly.  To address your suggestion, we have re-evaluated the paper's organization and recognize the value of a more structured approach. We will revise the paper to include the following sections: "Research Questions," "Experiment Design," "Experiment Setup," and "Experiment Results.” However, we would like to point out that the content of "Experiment Design" and "Experiment Setup" will remain as it is.  It is essential to retain the detailed discussion of the research questions, experiment design, and experiment setup in their current positions within the paper. This arrangement maintains a logical flow and ensures that readers can seamlessly follow the connection between the research questions, the proposed approach, the experimental design and the experimental setup. By doing so, we aim to provide a coherent narrative that guides the reader through the study's objectives and methodology before delving into the specific results.  Comments regarding qualitative analysis is detailed a few cells below this.  Comments regarding “additional details, and a broader experimental evaluation as well as exploration of modern techniques to enhance its comprehensiveness and impact.” are detailed and addressed in the revised manuscript. | Rewrote introduction to be made more concise. Separated the first part of initial “Proposed approach and experimental setup” into 2 sections – “experimental design” and “experimental setup”. |
| Reviewer #1: The current introduction is overly lengthy, containing background information that can be moved to a separate background section. The introduction should instead focus on summarizing the proposed approach, existing problems in related work, the contributions of the paper, and the structure of the paper. | Introducing the research gaps in the introduction is rather important in explaining the proposed framework. Though we do agree it is lengthy and can be further made concise. | Revised and rephrase most of the paragraphs in introduction to improve conciseness and clarity especially on the objectives of the paper. |
| Reviewer #1: The acknowledgement section contains pseudo text which should be removed or corrected. | We sincerely apologize for the careless mistake. Changes have been made to address this issue. | This section has been removed. |
| Reviewer #1: The abstract is vague regarding the use of cross-project code clones in the proposed approach. The authors need to clarify this, specifically detailing its relationship with identifying software artifacts suitable for reuse. | Thanks for the suggestion. We have revised the abstract in the revised manuscript to address the comment. | Rewrote the abstract adding slightly more detail to that portion. |
| Reviewer #1: Figure 1 lacks informativeness and could be omitted. | Figure 1 initially was to describe how classification and regression is done with the context of software reuse in visual form. It is true that it might be unnecessary. | We have removed Fig 1 in the revised manuscript. |
| Reviewer #1: A software system may include many different software artifacts. Thus, the authors need to precisely specify for what software artifacts they want to predict the reusability. | Thank you for your comment. We appreciate the opportunity to clarify our approach regarding the software artifacts used in our study. We use the phrase ‘Maven artefact’ to refer to the entire project repository. An example would be <https://mvnrepository.com/artifact/junit/junit>. We analyse only the latest version at the time during the experiment was conducted. The Maven website itself uses the word ‘artifact’ to refer to each entry.  Our paper addresses the granularity of the artifacts analyzed. Specifically, we focus on **method**, **class**, and **file** levels of the artefact. 3 artefact-level metrics were also added as part of the analyses. All this information is detailed in section 4.1. | - |
| Reviewer #1: Several technical details of the proposed approach are missing. For instance, the list of features in Section 3.2.7, the justification for selecting K=4 in K-fold validation, and the reasoning for choosing only two levels (High and Low) for reusability and not including a Moderate level. | - | The 4-fold validation is explained and addressed.  Choosing 2 levels for reusability is also explained and addressed in the paper. |
| Reviewer #1: The evaluation section misses an empirical study to gauge user perceptions of the proposed approach. Including interviews with expert developers to identify important features and comparing their feedback with the paper’s findings could provide valuable insights   Reviewer #1: Currently, the paper provides the quantitative results of the experiments but lacks the qualitative results. The quantitative results need to be qualitatively analyzed to offer better insights | Thank you for your valuable feedback. We understand the importance of providing a qualitative perspective to complement the quantitative results. However, we would like to clarify that adding qualitative aspects (i.e., getting expert opinions) will deviate the focus of our paper away from the main objectives. One of the paper’s objectives is to address the gap of contradicting or multiple opinions from experts or developers similar to Papamichail’s (2019) proposal. Part of our paper addresses the limitations of expert reviews in evaluating software reuse, pointing out that such reviews can be subject to human bias. The primary motivation of our research is to advocate for the use of machine learning to automate this process, providing a more objective, consistent, and scalable solution for predicting software reuse. By minimizing human intervention, our approach reduces the risk of bias, making the prediction process more reliable. This paper’s quantitative experiment has proven that it is possible to address the issue. Conducting another qualitative study on the results might potentially jeopardise focus of the paper.  We hope this clarification addresses your concerns, and we believe the paper has provided meaningful and sufficient insights in alignment with the quantitative nature of the data. | - |
| Reviewer #1: The paper primarily uses traditional machine learning techniques as mentioned in Section 3.3. It would be beneficial to explore newer deep learning or modern LLM-based techniques, as these have shown promising results in various software engineering tasks. | Thank you for this insightful feedback. We agree that deep learning and LLM-based techniques have shown significant promise in various software engineering tasks. However, our research uses numerical statistical-based data, where traditional machine learning techniques are particularly more effectiveness for this type of data.  The use of deep learning or LLMs, while innovative, might introduce unnecessary complexity and computational overhead given the nature of our data and the specific goals of our study. Deep learning models are beneficial when there is high volume of data. For example, Jyothi (2022) and Hameed (2021) has demonstrated that deep leaning outperforms traditional ML in image data. As for LLMs, these models are complex and are mostly used with text data (Pathil et. al., 2024), which is out of scope of this study. Traditional methods provide a clear and efficient approach to addressing the research questions posed.  We acknowledge that exploring more advanced techniques could be a valuable direction for future work. For now, our approach is designed to explore and leverage the strengths of traditional methods in the context of our research objectives and data characteristics. Another key goal of this research is to explain how ML helps in identifying factors that impact software reuse. Using LLMs or deep learning techniques may impede this goal’s progress as it will take a significant computation and resources to achieve such goal. Lastly, deep learning and LLM-based methods also fall outside the scope of this research, as our focus is on practical, interpretable, and efficient solutions tailored to the characteristics of the data we are working with. | - |
| Reviewer #2: Explain in more detail the limitations of your approach for example Java only projects. | Thank you for your insightful comment. We appreciate the opportunity to clarify the limitations of our approach, particularly with regard to its focus on Java-only projects.  Our research primarily utilizes Java-based projects for several reasons, which also highlight certain limitations:   1. **Scope Limitation**: By focusing exclusively on Java projects, our study is constrained to the characteristics and conventions of the Java programming language. This choice is driven by the availability of well-established data sources and tools (e.g., Maven artifacts and GitHub projects) specific to Java. As a result, the findings and models derived from this research are directly applicable to Java-based software but may not generalize to projects written in other programming languages. 2. **Tool and Metric Specificity**: The metrics and tools used for this study, such as SourceMeter, are tailored to Java codebases. The metrics and their aggregation methods might differ in other languages, which could impact the applicability of our results to non-Java projects. For example, certain Java-specific metrics (e.g., those related to Java's object-oriented features) may not have direct counterparts in languages with different paradigms. 3. **Generalizability**: While the Java-based approach provides valuable insights into software reuse within the Java ecosystem, it may not capture the nuances of software reuse in other ecosystems, such as those based on C++, Python, or JavaScript. Different languages have distinct idioms, libraries, and reuse practices, which could influence the applicability of our findings to these contexts. 4. **Data Availability**: The choice to focus on Java projects is also influenced by the availability of comprehensive and high-quality data from popular repositories like Maven and GitHub. For other languages, obtaining similar datasets with consistent quality and size may present challenges.   We acknowledge that these limitations are important for understanding the scope and applicability of our findings. We hope this explanation clarifies the constraints of our approach and provides context for the specific focus on Java projects in our study. | Made some amendments in the 2^nd^ paragraph of the threads to validity section. |
| Reviewer #2: Move figures and tables closer to where they are first referenced. | - | Figures and tables were move closer to where they were referenced. |
| Reviewer #2: Please test the application at <https://reuse-assessment.streamlit.app/Evaluate> with invalid input such as empty strings. When I did that, the application did not gracefully failed and did not give an appropriate error. | Thanks for pointing this out. | Handling empty input has been fixed. |
| Reviewer #2: Please make your dataset available publicly for reproducibility and benefit of other researchers. | Dataset link has been publicly available. It is attached as a footnote. Under Section 3.2.1.  <https://github.com/cyan-wings/software-reuse-thesis/blob/master/Classification/Dataset.csv> | - |
| Reviewer #2: English corrections: 1. 'The ground truth of software reuse ...'. 2. '40% of reused code'. 3. 'such a manual approach is not readily available and is expensive'. 4. 'an existing software'. 5. Use correct commas in 'utilisation of code developed by third parties'. 6. 'an artefact' 7. Use correct commas around "35" 8. Correct commas around 'Maven Usages'. 9. Correct commas around 'Usages'. 10. 'Yellow line' appears 'red line' in Fig. 6. 11. Duplicate sentence: 'Cross-validation splits are performed at this stage to avoid data leakage'. 12. Shouldn't the acronyms be all uppercase? KBF, RFI? 13. Put equation (3) after it is first referenced, and not before. 14. In Fig. 7 caption, should it be 'R' or 'RR? In the previous references you use 'R' but here you are using 'RR'. Be consistent. 15. 'Practitioners can use these pipeline methods ...' 16. Appendix number is missing in last line on Page 39. 17. '... these findings further validate the advantage ...' 18. Appendix number missing on Line 1082, Page 42. 19. Provide a mathematical formula for this computation: 'Table 7 shows the top important features after aggregating the feature importance score weighted by the F1-score of the cross-validation instance and the overall F1-score of the model'. 20. The section under Acknowledgments has garbage text. Please remove it or put an actual acknowledgement. | Thank you for the detailed corrections and also the request for more details on some of the sections. | Updated Fig 6.  Added appendices which were initially missing.  Added equation above table 7 to make the importance calculation clearer. |

With the above outline, we have made significant changes and hope to have provided sufficient details to the article. We would also wish to clarify in case of any misunderstandings following this rebuttal.

Regards,

Matthew Yeow.

# References

1. Papamichail, M. D., Diamantopoulos, T., & Symeonidis, A. L. (2019). Measuring the reusability of software components using static analysis metrics and reuse rate information. *Journal of Systems and Software*, *158*, 110423. doi:10.1016/j.jss.2019.110423.
2. Jyothi, P., Singh, A.R. Deep learning models and traditional automated techniques for brain tumor segmentation in MRI: a review. *Artif Intell Rev* **56**, 2923–2969 (2023). <https://doi.org/10.1007/s10462-022-10245-x> .
3. Hameed BMZ, Prerepa G, Patil V, et al. Engineering and clinical use of artificial intelligence (AI) with machine learning and data science advancements: radiology leading the way for future. *Therapeutic Advances in Urology*. 2021;13. doi:[10.1177/17562872211044880](https://doi.org/10.1177/17562872211044880) .
4. Patil R, Gudivada V. A Review of Current Trends, Techniques, and Challenges in Large Language Models (LLMs). *Applied Sciences*. 2024; 14(5):2074. <https://doi.org/10.3390/app14052074> .
